# Lifestyle factors as mediators of area-level socioeconomic differentials in mental health and cognitive function: the Tromsø Study

**DOI:** 10.1136/jech-2023-220928

**Published:** 2023-11-22

**Authors:** Sweta Tiwari, Ester Cerin, Tom Wilsgaard, Ola Løvsletten, Sameline Grimsgaard, Laila Arnesdatter Hopstock, Henrik Schirmer, Annika Rosengren, Kathrine Kristoffersen, Maja-Lisa Løchen

**Affiliations:** 1 Department of Community Medicine, UiT The Arctic University of Norway, Tromsø, Norway; 2 Department of Clinical Medicine, UiT The Arctic University of Norway, Tromsø, Norway; 3 Institute for Health and Ageing, Australian Catholic University, Melbourne, Victoria, Australia; 4 Department of Health and Care Sciences, UiT The Arctic University of Norway, Tromsø, Norway; 5 Department of Cardiology, Akershus University Hospital, Lorenskog, Norway; 6 Institute of Clinical Medicine, University of Oslo, Oslo, Norway; 7 Department of Molecular and Clinical Medicine, University of Gothenburg, Gothenburg, Sweden; 8 Department of Health and Care, Tromsø Municipality, Tromsø, Norway

**Keywords:** cognition, depression, epidemiology, health inequalities, mental health

## Abstract

**Introduction:**

Low socioeconomic status (SES) is associated with poor mental health and cognitive function. Individual-level SES and area-level SES (ASES) may affect mental health and cognitive function through lifestyle. We aimed to quantify the associations of ASES with mental health and cognitive function and examine the mediating role of lifestyle behaviours independent of individual-level SES in a Norwegian population.

**Methods:**

In this cross-sectional study, we included 7211 participants (54% women) from the seventh survey of the Tromsø Study (2015–2016) (Tromsø7). The exposure variable ASES was created by aggregating individual-level SES variables (education, income, housing ownership) from Statistics Norway at the geographical subdivision level. Tromsø7 data were used as mediators (smoking, snuff, alcohol, physical activity, diet) and outcomes (cognitive function, anxiety, depression, insomnia). Mediation and mediated moderation analysis were performed with age as a moderator, stratified by sex.

**Results:**

Higher ASES was associated with better cognitive function and fewer depression and insomnia symptoms, independent of individual-level SES. These associations were mediated by smoking and physical activity. Alcohol was a mediator for depression and cognitive function in women. Age was a significant moderator of the association between ASES and global cognitive function in women. The largest total indirect effect of ASES was found for depression, with the joint effect of the mediators accounting for 36% of the total effect.

**Conclusions:**

People living in areas with lower ASES are at higher risk of poor mental health, such as depression and insomnia, and have lower cognitive function possibly due to unhealthy lifestyle (smoking, alcohol and physical inactivity).

**WHAT IS ALREADY KNOWN ON THIS TOPIC**

- Individual-level and area-level socioeconomic status (ASES) are associated with mental and cognitive health.
- There is a dearth of findings on the potential mediating role of lifestyle factors in the ASES-mental/cognitive health association (independent of individual SES) in the general population.

**WHAT THIS STUDY ADDS**

- Lower ASES is associated with lower cognitive function, high risk of depression and insomnia symptoms independent of individual-level SES.
- The risk is mediated by unhealthy lifestyle behaviours such as smoking, physical inactivity, high alcohol intake and low fruit and vegetables consumption.
- Lifestyle behaviours were examined as single, separate mediators as well as conjoint mediators. This allowed for estimation of the total contribution of all examined lifestyle behaviours to the associations between ASES and mental health and cognitive function.

**HOW THIS STUDY MIGHT AFFECT RESEARCH, PRACTICE OR POLICY**

- Targeted public health interventions aimed at reducing area-level inequalities are essential to improve health in the population, which can be attained by targeting the behavioural pathways responsible for the link between ASES and mental/cognitive health.

## Background

Mental ill health, such as anxiety, depression, insomnia and cognitive impairment, are characterised by impaired cognition, emotional regulation and behaviour.^1^ Surpassing both cardiovascular diseases and cancer, mental and neurological disorders and substance abuse constitute 13% of the global burden of disease (GBD).^2^ Depression is the third leading contributor to GBD.^2^ Insomnia is a neglected condition with a prevalence of 10–30%.^3^ Long-term insomnia may lead to depression, impaired work performance and poor quality of life.^3^ More than 46 million people suffer from dementia, and this number is expected to increase.^4^ Even mild cognitive impairment (MCI) causes significant disability and medical costs.^5^ As no effective treatment for dementia and MCI is available, risk reduction is the best strategy which includes cognitive health promotion throughout adulthood by targeting relevant modifiable lifestyle risk factors.^6^

Socioeconomic status (SES) is a consistent correlate of mental health and cognitive function, with lower SES linked to higher prevalence of mental illness.^7^ Individuals with lower SES in terms of educational attainment, income and occupation tend to have poorer mental health and cognitive function.^8–10^ The physical and social characteristics of where people live, particularly neighbourhood SES or area-level SES (ASES), are associated with mental health and cognitive function independently of individual-level SES.^11–14^ Differences in lifestyle based on individual-level SES and ASES are likely responsible for the observed associations. Lifestyles, mainly smoking and excessive alcohol use, are important intermediary factors in the causal pathway between low individual-level SES and poor health.^15^ Moreover, diet, physical activity, smoking and alcohol influence mental health.^16^ Previous studies have found that ASES is associated with lifestyle behaviours independent of individual-level SES.^17–20^ A cross-sectional study showed an association between the neighbourhood physical environment and cognitive health, with a mediating role of physical activity.^21^

ASES may affect mental health and related lifestyle independent of individual-level SES through the exposure to the behaviours of other individuals residing in the same area.^22^ This ‘neighbourhood effect’ can be described through social interactive mechanisms, according to which an individual’s behaviours, aspirations and attitudes change or are influenced as the result of social contacts with neighbours or to conform to local social norms.^23^ One of the most influential pathways of neighbourhood effects on health is the health behaviour pathway from Meijer’s model, which refers to the ways neighbourhoods may affect health behaviours that are closely linked to disease and mortality.^24^ Our study focuses on this pathway, where we estimate the association of ASES with mental health and cognitive function and the mediating role of lifestyle in this association.

While studies have reported associations between ASES and mental health and cognitive function,^11–14^ only few have examined lifestyle factors as mediators of the associations in a general population. This study aimed to quantify the associations of ASES with mental health problems and cognitive function and examine if these associations are mediated by lifestyle behaviours (smoking, snuff, alcohol, physical activity and diet) independent of individual-level SES in a general population. We hypothesised that ASES would be associated with better mental health and cognitive outcomes and that these associations would be due to leading a healthier lifestyle. We examined the single as well as the combined mediating effects of lifestyle behaviours. This allowed for estimates of the total contribution of all lifestyles to the associations between ASES and mental health and cognitive function. In addition, unlike other studies, this study was performed in a municipality with a rather large geographical area with the majority of the population being concentrated in the town centre and suburbs close by. Finding between-area differences in mental health outcomes and health-related behaviours within such a geographical context would have important implications for the formulation of policies and interventional strategies on a larger scale. The findings from this study can be useful in understanding the causal pathways through which ASES impacts mental health and cognitive function and targets the most important mediators in the association when developing interventional strategies. In addition, the findings from this study can inform the planning and development of area-oriented health promotion strategies and local interventions to improve mental health.

## Methods

### Study population

The Tromsø Study is a population-based study.^25^ Seven surveys (Tromsø1 to Tromsø7) were conducted from 1974 to 2016. To each survey, invitations to attend the examinations were sent by mail to registered inhabitants of the Tromsø municipality, including both full birth cohorts and/or random samples. Data collection includes questionnaires, biological sampling, and clinical examinations.

This study includes data from participants attending the second visit in Tromsø7 conducted in 2015–2016. All inhabitants 40 years and older (N=32 591) were invited to the first visit, and a subsample (N=13 028) was pre-marked to the second visit for extended comprehensive examinations including cognitive function tests. A total of 21 083 (65%) attended the first visit, and from the pre-marked sample 8346 participated in the second visit.^25^ After exclusion, 7211 women and men were included for the main analysis (online supplemental figure 1). For the subanalyses, 4871 participants with valid data from the Tromsø7 Food Frequency Questionnaire (FFQ) were included (online supplemental figure 1).

10.1136/jech-2023-220928.supp1 Supplementary data

### Study area

Tromsø municipality is the largest urban area in Northern Norway. Most of the population live in the city centre and nearby suburbs, and 20% lives in rural areas. The geographical distance between high and low SES areas is small, particularly in the urban part of the municipality. As per previous study and reports, Tromsø is divided into 36 subdivisions (hereafter referred to as ‘areas’).^26 27^ More details about the study area, including a map (online supplemental figure 2),^28^ can be found in the online supplemental material.

### Area-level SES (exposure)

Data on individual-level SES and geographical areas (36 areas) (2015) were collected from Statistics Norway. Individual-level SES variables included individual and household income (after tax), education and house ownership. Individual income included work income, cash for care and parental benefit. Education was categorised into five levels as unknown or no education, elementary school, upper secondary or vocational school, less than 4 years of university education and 4 or more years of university education. House ownership was categorised as rented versus own housing. Standardised individual-level SES variables were summed to obtain a composite individual-level SES index. The overall ASES index for each area was calculated as the mean of all individual-level SES Z-scores in each area (subdivision),^26^ with a range from −1.5 to 1.1. Higher values reflected higher ASES.

### Lifestyle behaviour measures (mediators)

Questionnaires were used to collect information about smoking (never/previous/occasional/current), snuff use (never/previous/current), alcohol use and physical activity level. Alcohol units per drinking session was assessed with the Alcohol Use Disorder Identification Test^29^ categorised as no alcohol/1–2 units/3–4 units/5 or more units. Physical activity was categorised as sedentary, light or moderate-to-vigorous activity with the Saltin and Grimby questionnaire.^30^ The FFQ-retrieved intakes in grams/day (g/day) of fruits, vegetables, saturated fat and sugar were calculated using the University of Oslo developed food database KBS AE14 and KBS software system.^31^

### Mental health and cognitive function (outcomes)

Information about depression, anxiety and insomnia symptoms were collected from questionnaires. Cognitive function was assessed by the 12-word memory test, Digit Symbol Coding test, tapping test and Mini-Mental State Examination (MMSE)^25^ (see online supplemental file (page 4)).

### Statistical analysis

Participants’ characteristics were presented as means and SD for continuous variables and proportion for categorical variables adjusted for age stratified by sex and ASES quartiles. Differences between ASES quartiles in outcomes and mediators were estimated by regression analysis. Direct acyclic graphs were created for visual representations of causal assumptions between variables (online supplemental figure 3). Generalised linear mixed models and generalised additive mixed models with random intercepts at the area level were used to examine the confounder-adjusted associations of ASES with mental health outcomes.

Mediation analysis was performed to estimate mediation and mediated moderation effects. The lifestyle factors used as mediators were smoking, snuff use, alcohol consumption and physical activity level. Foods and nutrients were used as mediators in the subanalyses. The outcomes were anxiety, depression, insomnia symptoms and cognitive function. All models included the standardised individual-level SES variable as a covariate. Age was examined as a moderator of the associations. Random slopes for age were added in the model to see if it improved the fit of the model (via likelihood ratio test). All analyses were sex stratified. The analyses are described in the online supplemental file (page 6–7). Our use of the term ‘effect’ is purely statistical and does not imply causality.

## Results

Age-adjusted characteristics of women and men by ASES quartiles are presented in tables 1 and 2. The mean age was 62 years. Participants in areas with low ASES scores had lower cognitive function scores, lower fruit and vegetable intake, were more likely to be daily smokers, to be either a non-drinker or have higher intake of alcohol per drinking session, to be sedentary, to have higher scores for depression and insomnia symptoms (statistically significant in women only) compared with those in areas with high ASES scores.

**Table 1:** Age-adjusted characteristics of women by area-level socioeconomic status (ASES) score in quartiles: the Tromsø Study 2015–2016

Characteristics	ASES score in quartiles				P value for equality between ASES quartiles
	Lowest (n=1025)	Second (n=977)	Third (n=943)	Highest (n=945)	
Age, years	63.6 (10.0)	62.1 (10.7)	62.3 (10.2)	61.2 (10.4)	<0.0001
Verbal memory test*	7.3 (2.0)	7.6 (2.0)	7.6 (2.0)	7.7 (2.0)	<0.0001
Digit Symbol Coding test†	43.8 (12.1)	45.2 (11.9)	46.6 (16.0)	46.6 (12.6)	<0.0001
Tapping test‡	54.8 (8.7)	54.8 (8.6)	56.2 (8.3)	56.1 (8.4)	<0.0001
Mini-Mental State Examination§	27.8 (2.3)	28.1 (2.2)	28.3 (2.0)	28.2 (2.0)	<0.0001
Anxiety symptoms¶	3.3 (2.8)	3.4 (3.0)	3.2 (2.8)	3.2 (2.8)	0.45
Depression symptoms**	2.7 (2.6)	2.7 (2.7)	2.2 (2.4)	2.5 (2.5)	<0.0001
Insomnia symptoms††	10.0 (10.3)	10.1 (10.2)	9.0 (9.4)	8.8 (8.9)	0.0037
Alcohol (per drinking session), %					0.0001
No alcohol	11.3	8.4	5.5	6.2	
1–2 units	67.3	71.3	75.2	75.5	
3–4 units	19.1	18.4	17.4	16.3	
5 or more units	2.2	1.9	1.9	2.0	
Smoking, %					0.0003
No smoking	32.1	36.0	38.1	39.0	
Previous smoking	49.8	47.5	49.2	47.9	
Occasional smoking	2.3	2.9	3.0	3.2	
Daily smoking	15.9	13.6	9.8	9.8	
Snuff use, %					0.23
No snuff user	95.3	96.3	96.9	96.3	
Previous snuff user	1.7	1.9	1.8	2.0	
Current snuff user	3.0	1.8	1.3	1.7	
Physical activity, %					<0.0001
Sedentary	14.2	13.5	10.5	10.3	
Light activity	69.1	69.5	65.6	66.1	
Moderate-to-vigorous activity	16.8	16.9	23.8	23.6	
**Foods/nutrients**	**Lowest** (**n=665**)	**Second** (**n=675**)	**Third** (**n=629**)	**Highest** (**n=638**)	
Fruits and vegetables (g/day)	570 (328)	619 (314)	615 (324)	611 (286)	0.02
Saturated fat (g/day)	31 (14)	31 (13)	30 (13)	30 (12)	0.89
Sugar (g/day)	28 (27)	27 (29)	25 (25)	27 (26)	0.41

P value for linear trend.

*Scores are given as the number of correct words recalled (0, 12).

†Scores are given as the number of correct symbols coded (0, 96).

‡Scores are given as the average number of taps in 10 s.

§Scores are given as the number of correct memory (0, 30).

¶Anxiety score (0–21).

**Depression score (0–21).

††Insomnia score (0–42).

ASES, area-level socioeconomic status.

**Table 2:** Age-adjusted characteristics of men by area-level socioeconomic status (ASES) score in quartiles: the Tromsø Study 2015–2016

Characteristics	ASES score in quartiles				P value for equality between ASES quartiles
	Lowest (n=869)	Second (n=860)	Third (n=828)	Highest (n=764)	
Age, years	63.7 (10.1)	63.1 (10.6)	62.8 (10.5)	61.4 (10.2)	0.0001
Verbal memory test*	6.8 (1.9)	6.9 (1.9)	7.2 (2.0)	7.3 (2.0)	<0.0001
Digit Symbol Coding test†	39.2 (11.5)	40.4 (14.2)	42.2 (11.7)	42.6 (11.7)	<0.0001
Tapping test‡	58.2 (8.3)	59.1 (8.2)	60.3 (8.1)	60.0 (8.1)	<0.0001
Mini-Mental State Examination§	27.6 (2.1)	27.7 (2.0)	28.0 (2.0)	28.1 (1.8)	<0.0001
Anxiety symptoms¶	2.6 (2.5)	2.6 (2.4)	2.5 (2.3)	2.7 (2.5)	0.24
Depression symptoms**	3.0 (2.6)	3.2 (2.8)	2.7 (2.6)	2.9 (2.5)	0.012
Insomnia symptoms††	6.9 (8.5)	6.6 (8.4)	6.5 (8.6)	6.9 (8.5)	0.72
Alcohol (per drinking session), %					<0.0001
No alcohol	5	4.8	3	3.2	
1–2 units	52.1	58.5	60.3	57.7	
3–4 units	30.7	25.6	29.9	31.5	
5 or more units	12.2	11.1	6.9	7.5	
Smoking, %					<0.0001
No smoking	26.5	33.1	36.3	38	
Previous smoking	55	50.5	52.8	47.5	
Occasional smoking	3.3	3.9	3.3	5.8	
Daily smoking	15.2	12.5	7.7	8.8	
Snuff use, %					0.19
No Snuff user	83.1	86	85	83.7	
Previous snuff user	7.8	7.4	6.9	9.3	
Current snuff user	9.1	6.6	8.1	6.9	
Physical activity, %					<0.0001
Sedentary	17.5	13.8	12.2	10.6	
Light activity	56.8	53.5	51.4	53.7	
Moderate-to-vigorous activity	25.7	32.7	36.4	35.7	
**Foods/nutrients**	**Lowest (n=575**)	**Second (n=570**)	**Third (n=600**)	**Highest (n=519**)	
Fruits and vegetables (g/day)	484 (314)	492 (305)	539 (352)	549 (341)	0.001
Saturated fat (g/day)	35 (14)	36 (14)	35 (14)	34 (13)	0.17
Sugar (g/day)	32 (30)	33 (30)	32 (29)	30 (28)	0.34

P value for linear trend.

*Scores are given as the number of correct words recalled (0, 12).

†Scores are given as the number of correct symbols coded (0, 96).

‡Scores are given as the average number of taps in 10 s.

§Scores are given as the number of correct memory (0, 30).

¶Anxiety score (0–21).

**Depression score (0–21).

††Insomnia score (0–42).

ASES, area-level socioeconomic status.

### Associations between ASES (exposure) and lifestyle behaviours (mediators) with age as moderator

ASES was inversely associated with the odds of being previous or current smoker compared with being non-smoker in both sexes (table 3). The association was moderated by age in women, with stronger effects in older participants and a non-significant association in younger. Higher ASES was associated with higher odds of consuming 1–4 units of alcohol per drinking session compared with consuming no alcohol and positively associated with physical activity in both sexes (table 3). The association was moderated by age and stronger in older participants (table 3). A significant positive association was found between ASES and fruit and vegetable intake in both sexes. The association was moderated by age in men, with stronger effects in older participants (table 3).

**Table 3:** Associations between area-level socioeconomic status (exposure) and lifestyle behaviours (mediators) by sex and as main effects or effects moderated by age (n=7211): the Tromsø Study 2015–2016

Mediators	Main effects not moderated by age		Effects moderated by age					
	Women	Men	Women			Men		
	OR (95% CI)	OR (95% CI)	OR (95% CI)			OR (95% CI)		
			−1 SD	Mean age	+1 SD	−1 SD	Mean age	+1 SD
Smoking (ref: Never)								
Previous*		**0.77 (0.68 to 0.88**)	1.04 (0.90 to 1.20)	**0.88 (0.79 to 0.98**)	**0.75 (0.64 to 0.87**)	No significant interaction with age		
Occasional*		1.14 (0.86 to 1.51)	**1.66 (1.01 to 2.73**)	1.20 (0.87 to 1.64)	0.87 (0.61 to 1.23)			
Current*		**0.57 (0.46 to 0.69**)	0.82 (0.65 to 1.05)	**0.65 (0.54 to 0.78**)	**0.52 (0.41 to 0.65**)		–	–
Alcohol (ref: 0 units)	–						–	–
1–2 units*		**1.50 (1.14 to 1.96**)	**1.81 (1.45 to 2.25**)	**1.75 (1.42 to 2.17**)	**1.70 (1.18 to 2.45**)	No significant interaction with age		
3–4 units*		**1.48 (1.11 to 1.98**)	**2.10 (1.56 to 2.84**)	**1.64 (1.27 to 2.11**)	1.27 (0.86 to 1.89)			
5 or more*		0.96 (0.69 to 1.33)	2.18 (0.98 to 4.86)	**1.70 (1.01 to 2.88**)	1.33 (0.80 to 2.22)			
Snuff	–	0.97 (0.82 to 1.14)†	0.78 (0.45 to 1.37)†	0.82 (0.58 to 1.17)†	0.86 (0.65 to 1.14)†	No significant interaction with age	–	–
Never								
Previous								
Current								
Physical activity	–	–	1.06 (0.90 to 1.24)†	**1.28 (1.13 to 1.45)†**	**1.54 (1.31 to 1.82)†**	1.08 (0.91 to 1.29)†	**1.31 (1.14 to 1.50**)†	**1.58 (1.32 to 1.89**)†
Sedentary								
Light activity								
Moderate-to-vigorous activity								
**Dietary variables (women n=2607, men n=2264**)								
	**B (95%CI)**	**B (95%CI)**	**B (95%CI)**			**B (95%CI)**		
Fruits and vegetables (g/day)	**26.77 (5.91 to 48.41**)	–	No significant interaction with age	–	–	22.38 (−6.49 to 51.26)	**43.59 (22.98 to 64.21**)	**64.8 (34.89 to 94.7**)
Saturated fat (g/day)	−0.18 (−0.98 to 0.63)	−0.74 (−1.75 to 0.25)	No significant interaction with age	–	–	No significant interaction with age	–	–
Sugar (g/day)	−0.77 (−2.29 to 0.75)	−1.03 (−3.15 to 1.07)	No significant interaction with age	–	–	No significant interaction with age	–	–

All models are adjusted for age and individual-level socioeconomic status and accounted for area-level clustering. All effects are estimated per unit increase in ASES; OR=odds ratio; CI=confidence interval; SD=standard deviation; B=regression coefficient; ref=reference category; proportional odds assumption was tested to see if the variable is nominal or ordinal.

Bold typeface indicates significance.

*Nominal mediator, using generalised additive mixed models with multinominal variance.

†Treated as an ordinal variable (therefore only one value).

ASES, area-level socioeconomic status.

### Association between ASES (exposure) and mental health and cognitive function (outcome) with age as moderator

Higher ASES was associated with higher cognitive function scores in both sexes (table 4). One-unit increase in ASES was associated with 1.81 (95% CI 1.32 to 2.33) increase in the Digit Symbol Coding test score in women and 2.22 (95% CI 1.77 to 2.70) in men (table 4). Age was a significant moderator of the association of ASES with cognitive function as measured by the MMSE in women (table 4), where the effect was stronger in younger participants. Adding random slopes for age to the model did not change the result. Higher levels of ASES were associated with a decrease in depression and insomnia (women) symptoms. A unit increase in ASES was associated with a 0.25 (95% CI −0.36 to –0.13) unit decrease in depression symptoms score in women and 0.15 (95% CI −0.29 to –0.02) in men (table 5). Similarly, a unit increase in ASES was associated with a 0.81 (95% CI −1.28 to –0.33) unit decrease in insomnia symptoms in women (table 5).

**Table 4:** Results of mediation analysis for the total sample, women (n=3890) and men (n=3321): total, direct and indirect effects of area-level socioeconomic status on cognitive function (outcomes): the Tromsø Study 2015–2016

Effects	Verbal memory test		Digit Symbol Coding test		Tapping test		Mini-Mental State Examination	
	Women	Men	Women	Men	Women	Men	Women	Men
	B (95% CI)	B (95% CI)	B (95% CI)	B (95% CI)	B (95% CI)	B (95% CI)	B (95% CI)	B (95% CI)
Total effect*	**0.28 (0.19 to 0.38**)	**0.32 (0.23 to 0.41**)	**1.81 (1.32 to 2.33**)	**2.22 (1.77 to 2.70**)	**1.06 (0.69 to 1.41**)	**1.23 (0.89 to 1.57**)		**0.28 (0.18 to 0.37**)
Association at:†								
−1 SD	No significant interaction with age	No significant interaction with age	No significant interaction with age	No significant interaction with age	No significant interaction with age	No significant interaction with age	**0.40 (0.26 to 0.55**)	No significant interaction with age
Mean age							**0.28 (0.17 to 0.39**)	
+1 SD							**0.16 (0.02 to 0.31**)	
Direct effect‡	**0.27 (0.17 to 0.36**)	**0.29 (0.20 to 0.39**)	**1.74 (1.15 to 2.28**)	**2.01 (1.58 to 2.53**)	**0.89 (0.50 to 1.22**)	**1.11 (0.76 to 1.46**)		**0.27 (0.16 to 0.36**)
Association at:†								
−1 SD	No significant interaction with age	No significant interaction with age	No significant interaction with age	No significant interaction with age	No significant interaction with age	No significant interaction with age	**0.38 (0.24 to 0.53**)	No significant interaction with age
Mean age							**0.27 (0.16 to 0.38**)	
+1 SD							**0.15 (0.004 to 0.30**)	
Indirect effect, combined§	0.009 (−0.01 to 0.05)	0.03 (−0.001 to 0.05)	0.08 (−0.06 to 0.29)	**0.21 (0.02 to 0.33**)	**0.16 (0.07 to 0.31**)	**0.12 (0.01 to 0.23**)	0.01 (−0.01 to 0.04)	0.02 (−0.01 to 0.04)
PME¶	0.03 (−0.08 to 0.15)	0.09 (0.01 to 0.18)	0.04 (−0.05 to 0.16)	0.09 (0.03 to 0.17)	0.15 (0.04 to 0.29)	0.10 (0.01 to 0.20)	0.05 (−0.05 to 0.17)	0.06 (−0.03 to 0.17)
Exposure-adjusted effects of mediators:								
Smoking (ref: never)	No mediation	Mediation	Mediation	Mediation	Mediation	Mediation	No mediation	No mediation
Previous	0.005 (−0.12 to 0.13)	−0.12 (−0.26 to 0.02)	−0.01 (−0.76 to 0.74)	**−1.03 (−1.81 to to 0.25**)	0.15 (−0.36 to 0.66)	−0.34 (−0.87 to 0.19)	0.03 (−0.11 to 0.17)	−0.06 (−0.21 to 0.08)
Occasional	0.22 (−0.14 to 0.59)	0.11 (−0.20 to 0.43)	−1.92 (−4.03 to 0.21)	−0.61 (−2.40 to 1.18)	**−1.69 (−3.11 to to 0.24**)	−0.86 (−2.09 to 0.38)	0.10 (−0.34 to 0.53)	−0.09 (−0.43 to 0.25)
Current	−0.04 (−0.24 to 0.15)	**−0.35 (−0.56 to to 0.14**)	**−2.11 (−3.26 to to 0.96**)	**−2.33 (−3.54 to to 1.12**)	**−2.08 (−2.86 to to 1.29**)	**−2.15 (−2.98 to to 1.32**)	−0.09 (−0.31 to 0.13)	−0.07 (−0.30 to 0.15)
Alcohol (ref: 0 units)	Mediation	No mediation	No mediation	No mediation	Mediation	No mediation	Mediation	No mediation
1–2 units	**0.37 (0.15 to 0.59**)	0.07 (−0.24 to 0.38)	0.87 (−0.44 to 2.19)	1.44 (−0.34 to 3.24)	**1.16 (0.26 to 2.07**)	0.49 (−0.72 to 1.71)		0.18 (−0.14 to 0.51)
Association at:†								
−1 SD							**0.50 (0.21 to 0.76**)	
Mean age							0.15 (−0.12 to 0.42)	
+1 SD							−0.19 (−0.63 to 0.25)	
3–4 units	**0.35 (0.09 to 0.61**)	0.09 (−0.23 to 0.41)	0.77 (−0.72 to 2.29)	1.19 (−0.65 to 3.05)	**1.39 (0.36 to 2.43**)	0.21 (−1.04 to 1.48)		0.22 (−0.120 to 0.56)
Association at:†								
−1 SD							**0.53 (0.15 to 0.91**)	
Mean age							0.13 (−0.17 to 0.44)	
+1 SD							−0.26 (−0.72 to 0.20)	
5 or more	0.16 (−0.31 to 0.64)	0.02 (−0.35 to 0.38)	0.49 (−2.24 to 3.26)	0.25 (−1.83 to 2.33)	0.28 (−1.58 to 2.17)	0.16 (−1.25 to 1.58)		0.09 (−0.29 to 0.48)
Association at:†								
−1 SD							0.60 (−0.57 to 1.76)	
Mean age							0.20 (−0.52 to 0.93)	
+1 SD							−0.19 (−0.81 to 0.43)	
Snuff (ref: never)	No mediation	No mediation	No mediation	No mediation	No mediation	No mediation	No mediation	No mediation
Previous	−0.17 (−0.60 to 0.27)	0.12 (−0.10 to 0.35)	−0.80 (−3.33 to 1.70)	−0.69 (−2.00 to 0.62)	−0.08 (−1.81 to 1.64)	−0.14 (−1.04 to 0.77)	0.53 (−0.01 to 1.08)	0.20 (−0.04 to 0.45)
Current	0.11 (−0.32 to 0.55)	0.15 (−0.08 to 0.39)	−2.14 (−4.66 to 0.37)	**−1.85 (−3.21 to to 0.50**)	−1.09 (−2.81 to 0.63)	−0.70 (−1.62 to 0.22)	−0.08 (−1.04 to 0.88)	0.08 (−0.17 to 0.34)
Physical activity (ref: sedentary)	Mediation	Mediation	No mediation	Mediation	Mediation	Mediation	No mediation	No mediation
Light activity	**0.20 (0.02 to 0.39**)	0.16 (−0.02 to 0.35)	1.05 (−0.02 to 2.13)	**1.52 (0.47 to 2.56**)	**1.04 (0.32 to 1.78**)	**1.41 (0.70 to 2.12**)	0.11 (−0.09 to 0.31)	0.29 (0.09 to 0.48)
Moderate-to-vigorous activity	**0.23 (0.01 to 0.45**)	**0.22 (0.03 to 0.42**)	0.64 (−0.61 to 1.91)	**1.49 (0.36 to 2.61**)	**1.90 (1.04 to 2.76**)	**1.37 (0.60 to 2.13**)	0.04 (−0.19 to 0.28)	0.23 (0.02 to 0.44)

All models were adjusted for age and individual-level SES and accounted for area-level clustering.

Bold typeface indicates significance.

*Association between exposure and outcome: main effect presented when age is not a significant moderator; age-specific associations presented when age was a significant moderator.

†If significant interaction with moderator (age).

‡Association between exposure and outcome not explained by mediators.

§Association between exposure and outcome through mediators (dietary variables not included).

¶The proportion of the total effect mediated by mediator(s).

B, regression coefficient; CI, confidence interval; PME, proportion of mediated effect; SD, Standard deviation; SES, socioeconomic status.

**Table 5:** Results of mediation analysis for the total sample, women (n=3890) and men (n=3321): total, direct and indirect effects of area-level socioeconomic status on mental health (outcomes): the Tromsø Study 2015–2016

Effects	Anxiety symptoms		Depression symptoms		Insomnia symptoms	
	Women	Men	Women	Men	Women	Men
	B (95% CI)	B (95% CI)	B (95% CI)	B (95% CI)	B (95% CI)	B (95% CI)
Total effect*	−0.09 (−0.23 to 0.04)	0.05 (−0.07 to 0.18)	**−0.25 (−0.36 to 0.13**)	**−0.15 (−0.29 to 0.02**)	**−0.81 (−1.28 to 0.33**)	−0.09 (−0.56 to 0.36)
	No significant interaction with age	No significant interaction with age	No significant interaction with age	No significant interaction with age	No significant interaction with age	No significant interaction with age
Direct effect†	−0.07 (−0.22 to 0.05)	0.07 (−0.07 to 0.19)	**−0.16 (−0.29 to 0.04**)	−0.10 (−0.24 to 0.04)	**−0.65 (−1.21 to 0.24**)	0.01 (−0.46 to 0.47)
	No significant interaction with age	No significant interaction with age	No significant interaction with age	No significant interaction with age	No significant interaction with age	No significant interaction with age
Indirect effect, combined‡	−0.02 (−0.05 to 0.03)	−0.01 (−0.04 to 0.03)	**−0.09 (−0.13 to 0.03**)	**−0.05 (−0.09 to 0.02**)	−0.16 (−0.25 to 0.08)	−0.10 (−0.23 to 0.01)
PME§	0.26 (−1.92 to 2.67)	−0.26 (−3.47 to 3.39)	0.36 (0.16 to 0.75)	0.35 (0.06 to 1.76)	0.20 (−0.01 to 0.57)	1.07 (−5.93 to 6.35)
Exposure-adjusted effects of mediators:						
Smoking (ref: never)	Mediation	Mediation	Mediation	Mediation	No mediation	Mediation
Previous	0.20 (−0.003 to 0.40)	0.11 (−0.08 to 0.30)	0.03 (−0.15 to 0.21)	0.05 (−0.15 to 0.26)	0.21 (−0.49 to 0.91)	**0.73 (0.06 to 1.40**)
Occasional	0.38 (−0.18 to 0.94)	**0.73 (0.29 to 1.17**)	0.35 (−0.14 to 0.84)	0.16 (−0.32 to 0.64)	−0.17 (−2.09 to 1.75)	1.41 (−0.16 to 2.99)
Current	**0.56 (0.25 to 0.87**)	**0.30 (0.005 to 0.59**)	**0.81 (0.54 to 1.08**)	**0.33 (0.01 to 0.65**)	0.23 (−0.84 to 1.32)	0.06 (−0.98 to 1.10)
Alcohol (ref: 0 units)	No mediation	No mediation	Mediation	Mediation	No mediation	No mediation
1–2 units	0.09 (−0.27 to 0.44)	0.05 (−0.40 to 0.50)	**−0.61 (−0.93 to 0.30**)	−0.45 (−0.93 to 0.03)	−0.88 (−2.11 to 0.35)	0.51 (−1.03 to 2.05)
3–4 units	0.14 (−0.26 to 0.55)	0.09 (−0.37 to 0.56)	**−0.58 (−0.95 to 0.23**)	**−0.53 (−1.03 to 0.03**)	−0.06 (−1.47 to 1.36)	0.63 (−0.97 to 2.22)
5 or more	0.62 (−0.11 to 1.36)	0.20 (−0.31 to 0.72)	0.02 (−0.62 to 0.67)	−0.28 (−0.84 to 0.28)	1.77 (−0.77 to 4.31)	1.70 (−0.09 to 3.49)
Snuff (ref: never)	No mediation	No mediation	No mediation	No mediation	No mediation	No mediation
Previous	0.34 (−0.36 to 1.04)	0.26 (−0.05 to 0.58)	0.46 (−0.14 to 1.06)	0.32 (−0.03 to 0.66)	−0.11 (−2.45 to 2.45)	1.06 (−0.07 to 2.18)
Current	0.67 (−0.0004 to 1.34)	0.27 (−0.05 to 0.60)	**0.62 (0.04 to 1.22**)	−0.04 (−0.40 to 0.31)	**3.22 (0.92 to 5.53**)	**1.17 (0.01 to 2.33**)
Physical activity (ref: Sedentary)	No mediation	No mediation	Mediation	Mediation	Mediation	Mediation
Light activity	−0.07 (−0.37 to 0.22)	−0.13 (−0.39 to 0.12)	**−0.96 (−1.21 to 0.70**)	**−0.81 (−1.08 to 0.54**)	**−1.53 (−2.55 to 0.52**)	**−2.02 (−2.92 to 1.12**)
Moderate-to-vigorous activity	−0.12 (−0.47 to 0.22)	−0.26 (−0.53 to 0.01)	**−1.14 (−1.44 to 0.84**)	**−1.16 (−1.46 to 0.87**)	**−1.85 (−3.05 to 0.68**)	**−2.49 (−3.45 to 1.52**)

All models were adjusted for age and individual-level SES and accounted for area-level clustering.

Bold typeface indicates significance.

*Association between exposure and outcome: main effect presented when age is not a significant moderator; age-specific associations presented when age was a significant moderator.

†Association between exposure and outcome not explained by mediators.

‡Association between exposure and outcome through mediators (dietary variables not included).

§The proportion of the total effect mediated by mediator(s).

B, regression coefficient; CI, confidence interval; PME, proportion of mediated effect; SES, socioeconomic status.

### Mediators and mediated moderation of associations between ASES (exposure) and mental health and cognitive function (outcome)

Smoking mediated the association between ASES and cognitive function, with verbal memory test pertaining to current smoking in men. Current smoking appeared to be more important in the associations between ASES and psychomotor speed (tapping test) and attention and mental flexibility (Digit Symbol Coding test) in both sexes. However, ASES associations with global cognitive function (MMSE) were not explained by smoking status. Physical activity mediated the association between ASES and cognitive function as shown by verbal memory test, Digit Symbol Coding test and tapping test. The association between ASES and cognitive function as measured by MMSE was moderated by age, mediated by alcohol in women (table 4). Alcohol was a mediator with respect to verbal memory and psychomotor speed in women. Although ASES did not show a significant total effect on anxiety and insomnia symptoms (men), negative indirect associations were seen through smoking for anxiety and insomnia symptoms and through physical activity for insomnia symptoms in men (table 5). The association between ASES and depression symptoms was mediated by smoking, alcohol and physical activity in both sexes (table 5). Physical activity mediated the negative association between ASES and insomnia symptoms in women (table 5). The total mediated effect of ASES was significant for depression symptoms and cognitive function as measured by the tapping test for both sexes (indirect effect; tables 4 and 5). The largest total indirect effect of ASES (through all mediators together) was in relation to depression symptoms, with the mediators accounting for 36% of the observed effects (PME; table 5).

In the subgroup analyses (online supplemental table 1A and table 1B), including dietary variables, fruit and vegetables mediated the association between ASES and depression and insomnia symptoms in men. The largest total indirect effect of ASES was on depression symptoms, with the mediators accounting for 33% of the observed effects (PME; online supplemental table 1B).

## Discussion

In this population-based study, we found that higher ASES was associated with better health outcomes (cognitive function, depression and insomnia symptoms) independent of individual-level SES. These associations were mediated by lifestyle factors such as smoking and physical activity. Alcohol was a mediator for depression symptoms and certain aspects of cognitive function in women. Fruit and vegetable consumption was a mediator for depression and insomnia symptoms in men. Therefore, our hypothesis was supported by these findings, ASES was associated with better mental health and cognitive outcomes and these associations were due to leading a healthier lifestyle.

### ASES and health outcomes

Participants in neighbourhoods with higher SES had higher cognitive function and fewer depression and insomnia symptoms, independent of individual-level SES. Others have reported associations between individual SES and mental health and cognitive function.^8–10^ However, empirical evidence on associations between ASES and mental health and cognitive function independent of individual-level SES is limited. Our study suggests that ASES has a potential impact on mental health and cognitive function independent of individual-level SES. This can be explained by a social interactive mechanism and local social norms related to health behaviours that are relevant to mental health.^23^

Other studies found an association between low ASES and poor mental health.^14^ Recent cross-sectional studies reported that higher neighbourhood SES was associated with lower risk of dementia^12^ and better cognitive function.^32^ Various cross-sectional studies have also shown that living in a more deprived area was associated with depression.^11 33^ We also observed a negative association between higher ASES and insomnia symptoms in women. This has earlier only been shown on an individual-level SES.^34^ We did not find any association between ASES and anxiety, similar to another cross-sectional study.^33^ This might be because individuals with anxiety may be less involved in social interaction, with the neighbourhood environment exerting less influence on anxiety symptoms.

We also examined whether the association between ASES and health outcomes was moderated by age in sex-specific models. Age was a significant moderator of the association between ASES and global cognitive function in women as measured by the MMSE and stronger in younger participants. Inverse associations of ASES with depression and insomnia symptoms were found in both sexes and more in women. Women are more social and interact with neighbours more often than men.^35^ As a result of social interactive mechanisms, they may be more prone to changing their lifestyles as a result of this interaction, which may affect their mental health.^23^

### Lifestyle factors as mediators

We found associations between ASES and lifestyle factors, such as smoking, alcohol consumption, physical activity, and fruit and vegetables intake independent of individual-level SES. As noted earlier, this neighbourhood effect may be due to residents’ behaviour and well-being being influenced by their neighbours.^22^ Previous studies have also found that people with low ASES smoke more compared with those with higher ASES.^18^ Similar associations have been reported with respect to intake of alcohol,^19^ fruit and vegetables,^20^ and physical activity.^17^ ASES associations with physical activity might also be due to neighbourhood differences in the availability and quality of public spaces and recreational facilities.^36^

Lifestyle was also associated with cognitive function and symptoms of depression and insomnia. In general, healthier lifestyle was associated with better mental health, except for the association between alcohol and depression symptoms in both sexes and the association between alcohol consumption and certain cognitive functions in women. The protective effect of alcohol on depression and cognitive function could be due to low-to-moderate alcohol consumption being associated with social activities that may reduce depression symptoms^37^ and boost cognition.^21^ Another explanation could be that abstaining from alcohol could be due to poor health and/or medical advice not to drink alcohol due to medication use. Among the cognitive tests, the MMSE showed non-significant associations with several lifestyle factors. This might be due to ceiling effects associated with the MMSE. Specifically, most cognitively intact adults and older adults score high on the MMSE.^38^ The MMSE does not differentiate between different levels of cognitive function in an overall cognitively intact population as in our study sample.

We found that the associations between ASES and mental health and cognitive function were mediated by lifestyle factors as in few other studies.^21^ However, these studies did not examine the joint mediating effect of lifestyle behaviours. Additionally, unlike other studies, our study was performed in a municipality with a large geographical area with the majority of the population concentrated in the town centre and suburbs. Finding between-area differences in mental health outcomes and health-related behaviours within such a geographical context would have important implications for the formulation of policies and interventional strategies. Our findings suggest that targeted lifestyle interventions focusing not only on the individual but also on the neighbourhood level are important to improve an individual’s health.

### Strengths and limitations

This study is a large population-based study with high attendance including both sexes. We used validated questionnaires and standard measures to assess mediators and outcomes increasing the credibility of the questionnaires used in the study. A battery of sensitive tests feasible for population screening purposes was used for measuring cognitive function. These tests represent appropriate measures of cognition function in population-based studies decreasing the probability of missing true positive cases. The data for the exposure (ASES) were obtained from Statistics Norway, which provides complete official statistical data in Norway. The Norwegian unique personal identification number for every inhabitant allows exact matching of population register data, which was used to link data from Statistics Norway with the Tromsø Study. In contrast to self-administered questionnaires, the use of official statistical data prevents misclassification bias for the exposure variable in this study.

Limitations includes the cross-sectional design limiting causal inference. Changes in behaviours across time could have been examined. However, in Tromsø, ASES is quite stable over time, population mobility across areas is low and the effects of environmental exposures on mental health are likely to be cumulative and develop across many years. In addition, in the areas with greater internal migration, immigrants are more often from outside the municipality than across the municipal areas.^27^ We therefore performed cross-sectional analyses. Several mediators and outcome variables were self-reported. Misclassification bias could not be avoided as certain socially desirable habits tend to be over-reported and certain less acceptable habits are under-reported. Selection bias may occur, such as lower participation among individuals with dementia and mental ill health. There is a possibility of residual confounding as measures of individual-level SES and ASES do not encompass all factors that define SES such as occupational status, mortgages and family assets. External validity refers to a Caucasian population and may not be generalizable.

## Conclusion

People living in areas with lower ASES are at higher risk of having poor mental health and having lower cognitive function. This risk is mediated by modifiable lifestyle behaviours.

## Data Availability

Data may be obtained from a third party and are not publicly available. Data may be obtained from a third party and are not publicly available. The datasets presented in this article are not readily available because the data analysed in this study is subject to the following licenses/restrictions: The dataset is available upon application to the Tromsø Study and Statistics Norway.
