# Supplementary material for: Lifestyle factors as mediators of area-level socioeconomic differentials in mental health and cognitive function: the Tromsø Study
Source: J Epidemiol Community Health. 2023 Nov 22;78(2):88–97. doi: 10.1136/jech-2023-220928 (PMC10850631; doi:10.1136/jech-2023-220928)
Supplement: Supplementary data [file jech-2023-220928supp001.pdf]

Supplementary material

Lifestyle factors as mediators of area-level socio-economic differentials in mental health and cognitive function. The Tromsø Study

Tiwari S, *et al. J Epidemiol Community Health* 2024; 78:88–97. doi: 10.1136/jech-2023-220928

1. **Study Sample**

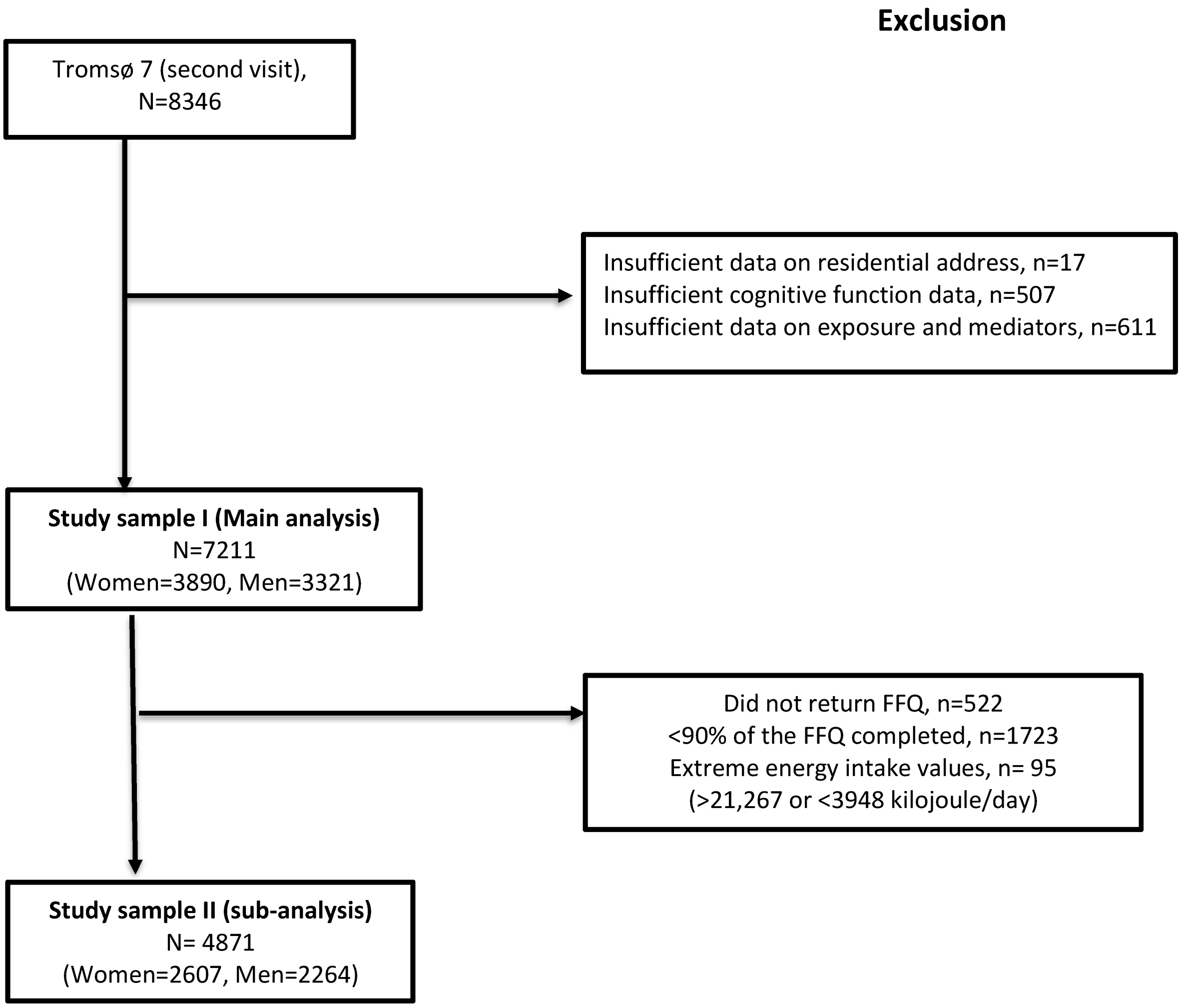

Figure 1: Study sample, The Tromsø Study 2015-2016. FFQ: food frequency questionnaire

2. Study areas of Tromsø municipality

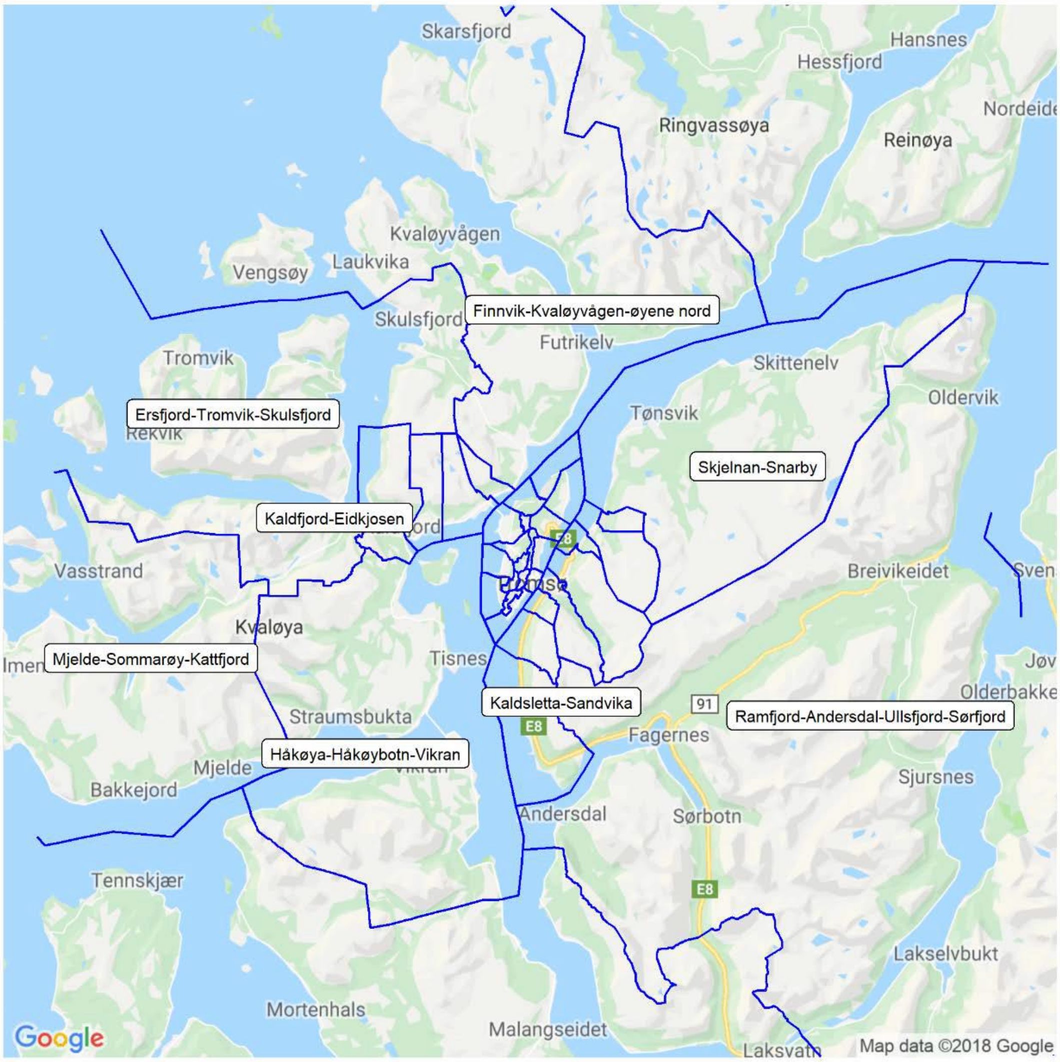

Figure 2: Map of Tromsø municipality with areas [1]. (Required permission to reuse the image was obtained)

Tromsø municipality had a total population of 72,066 in 2015. Only 9.3% of the population in Tromsø are non-western immigrants (Eastern Europe, Asia, Africa, and Latin America). Population mobility is not high in the municipality, and even in the areas with greater internal migration, immigrants are from outside the municipality rather than from within the municipality areas. The proportions of immigrants by areas are presented elsewhere [2]. The

36 areas used in this study were defined by the homogeneity of building type/living environments and the number of residents. The geographical units were created to be sufficiently large to produce statistically meaningful results and are meaningful for the municipality's planning and policymaking [2]. Previous population-based Tromsø Study has found differences in risk factors across residential areas within the municipality [3] with lifestyle mediating the associations between ASES and CVD risk factors [4].

3. Mental health and cognitive function variable measures

Depression, anxiety, and insomnia symptoms data were collected from self-administered questionnaire. Insomnia was defined according to Bergen Insomnia Scale [5]. Six items related to sleep were included in the questionnaire. Item scores represented number of days per week (0-7). The item scores were summed to create a total insomnia score (min: 0; max: 42). Anxiety and depression were assessed using the Hospital Anxiety and Depression Scale (HADS) [6]. The scale consists of 14 items, seven of which measure anxiety symptoms, while the remaining seven assess depressive symptoms. Item scores ranged from 0 to 3. The maximum total score was 21 for anxiety symptoms and 21 for depression symptoms.

Cognitive function was assessed by four different standardized tests. The twelve-word memory test is a test of short-term verbal memory with a immediate free recall of 12-word memory test. The words were shown written on a board and pronounced one at a time with 5 second intervals [7]. The participants were then given two minutes to recall the words. One point was given for each correctly recalled word, with the total score ranging from 0 to 12 points. The Digit-Symbol Coding test is a part of the Wechsler adult intelligence scale which is used to examine perceptual processing, speed, and memory Rows containing small blank squares were each paired with a randomly assigned number from one to nine. Above these rows, a printed key paired each number with a different symbol. The participants were asked to consecutively fill in with the corresponding symbol as many blank spaces as possible within 90 seconds. The tapping test is a test of psychomotor speed and lateralized coordination. The participants were asked to tap as many times as possible in 10 seconds with their index finger on a computer, which registered the number of taps. The task was repeated four times on both dominant and non-dominant hand. In the analyses, the mean of the average number of the three last taps on each hand was used [7]. The MMSE includes a global score of long-term verbal memory and episodic memory . The MMSE is divided into two sections, with the first part including oral responses and covering orientation memory and attention.

The maximum score is 21. The second part assesses a person's ability to name, follow verbal and written commands, copy a polygon, and write a spontaneous sentence. The maximum score for this part is nine. The total maximum score for the MMSE is 30.

4. Direct acyclic graph (DAG)

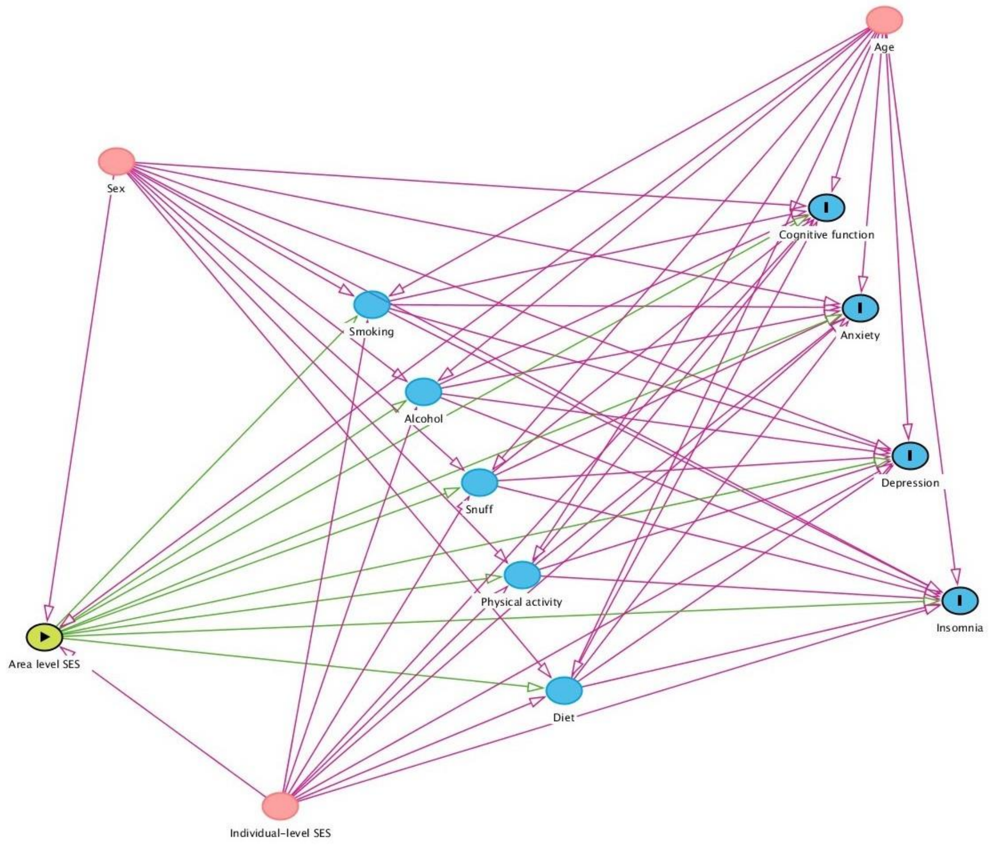

Figure 3: Directed Acyclic Graph illustrating exposures, outcomes, mediators, and confounders.

5. Stepwise description of mediation analysis

Analyses were conducted in four steps. We first estimated the associations between ASES (exposure; X in the regression equation below) with the outcomes (Y) adjusted for age (moderator; Mod) and individual SES (covariates; Co) (total effect GLMM; Step 1).
We then added an age by ASES interaction term to the total effect GLMM to estimate the moderating effects of age (step 2). Moderation effects with a p-value< 0.05 were considered statistically significant.
We examined mediation effects if age was not identified as a moderator in step 2, and mediated moderation effects if age was identified as a moderator. This was done by first regressing lifestyle factors (Mediator; M) onto ASES (X), covariates (Co) and the interaction term between age and ASES (X.Mod), if it was significant in step 2 (GAMM; step 3). The mediators (smoking, alcohol, snuff, and physical activity) had three or more ordered categorical levels. To establish whether they could be modelled using ordinal or nominal regression, the proportional odds assumption was tested. If the assumption was violated, the mediator variable was treated as nominal and, if the assumption was not violated, it was treated as ordinal.
In the final step, we regressed the outcomes (Y) onto ASES (X) and lifestyle factors (M), covariates (Co) and interaction terms of exposure (X.Mod) and mediators (M.Mod) by age (the moderator), if it was identified in step 2. The four steps are described by the following regression equations:

Step 1- effect of ASES on outcome (b_1)

$$Y= b_1(X) + b_2(Mod) + b_3(Co)$$

Step 2- moderators of effect of ASES on outcome (b_6)

$$Y= b_4(X) + b_5(Mod) + b_6(X.Mod) + b_7(Co)$$

Step 3- effect of ASES (b_8) and its moderators (b_{10}) on lifestyle factors (mediator)

$$M= b_8(X) + b_9(Mod) + b_{10}(X.Mod)^* + b_{11}(Co)$$

Tiwari S, *et al. J Epidemiol Community Health* 2024; 78:88–97. doi: 10.1136/jech-2023-220928

Step 4- exposure-adjusted effect of mediator (b_{13}) and its moderators (b_{16}) on outcome, and direct effect of ASES (b_{12}) and its moderator (b_{15}) on outcome

$$Y = b_{12}(X) + b_{13}(M) + b_{14}(Mod) + b_{15}(X.Mod)^* + b_{16}(M.Mod)^* + b_{17}(Co),$$

where $b_1,\ldots,b_{17}$ are regression coefficients and the interaction terms denoted by * are included when b_6 is statistically significant in step 2. Mediation and mediated moderation were confirmed using the joint-significance test. According to this test, mediation is confirmed if both b_8 and b_{13} regression coefficients are statistically significant. Mediated moderation is confirmed when both b_{10} and b_{13} regression coefficients are statistically significant and/or both b_8 and b_{16} are statistically significant [4, 8, 9]. We remark that some mathematical details have been omitted in the equations in step 1-4 (e.g., the equations for nominal and ordinal regression are not correctly represented from a mathematical standpoint here), as the purpose was to highlight how mediation and mediated moderation are identified in a non-technical way.

The mediation analysis was conducted in R version 4.0.4 using the packages ‘lme4’, ‘mgcv’ and ‘medflex’, all other analyses were performed using STATA V.16. The medflex package in R was used to calculate the combined mediation effect of all mediators together and the proportion mediated effect (PME) [10].

6. Result of mediation analysis

Table 1A. Results of mediation analysis for the sub-sample, women (n=2607) and men (n=2264): total, direct, and indirect effects of area-level socio-economic status on cognitive function (outcomes). The Tromsø Study 2015-2016.

**Effects**	**Verbal memory test**		**Digit symbol coding test**		**Tapping test**		**Mini mental state examination**	
	**Women**	**Men**	**Women**	**Men**	**Women**	**Men**	**Women**	**Men**
	B (95% CI)	B (95% CI)	B (95% CI)	B (95% CI)	B (95% CI)	B (95% CI)	B (95% CI)	B (95% CI)
Total effect[a]	**0.29 (0.18, 0.40)**<br><br>No significant interaction with age	**0.34 (0.23, 0.46)**<br><br>No significant interaction with age	**1.87 (1.24, 2.56)**<br><br>No significant interaction with age	**2.26 (1.71, 2.86)**<br><br>No significant interaction with age	**1.01 (0.59, 1.43)**<br><br>No significant interaction with age	**1.17 (0.73, 1.59)**<br><br>No significant interaction with age	**0.19 (0.08, 0.30)**<br><br>No significant interaction with age	**0.20 (0.09, 0.31)**<br><br>No significant interaction with age
Direct effect[b]<br>Association at:*	**0.28 (0.16, 0.38)**<br><br>No significant interaction with age	**0.32 (0.20, 0.43)**<br><br>No significant interaction with age	**1.82 (1.13, 2.56)**<br><br>No significant interaction with age	**2.04 (1.52, 2.70)**<br><br>No significant interaction with age	**0.85 (0.41, 1.27)**<br><br>No significant interaction with age	**1.03 (0.59, 1.49)**<br><br>No significant interaction with age	**0.18 (0.08, 0.30)**<br><br>No significant interaction with age	**0.17 (0.07, 0.30)**<br><br>No significant interaction with age
Indirect effect, combined[c]<br>PME[d]	0.004 (-0.02, 0.05)<br>0.01 (-0.12, 0.15)	0.02 (-0.006, 0.06)<br>0.07 (-0.03, 0.18)	0.05 (-0.13, 0.25)<br>0.03 (-0.07, 0.15)	0.21 (-0.03, 0.38)<br>0.09 (0.003, 0.19)	**0.16 (0.02, 0.31)**<br>0.16 (0.02, 0.36)	0.14 (-0.01, 0.25)<br>0.12 (0.004, 0.27)	0.01 (-0.03, 0.03)<br>0.04 (-0.14, 0.26)	0.02 (-0.02, 0.05)<br>0.13 (-0.05, 0.43)
Exposure-adjusted effects of mediators:								
Fruits and vegetables (g/day)	-0.007 (-0.08, 0.06)<br>No mediation	-0.02 (-0.09, 0.05)<br>No mediation	-0.27 (-0.71, 0.16)<br>No mediation	0.004 (-0.37, 0.39)<br>No mediation	-0.06 (-0.34, 0.22)<br>No mediation	0.19 (-0.08, 0.47)<br>No mediation	0.002 (-0.07, 0.08)<br>No mediation	-0.02 (-0.09, 0.05)<br>No mediation
Saturated fat (g/day)	0.02 (-0.05, 0.09)<br>No mediation	0.03 (-0.03, 0.11)<br>No mediation	-0.26 (-0.70, 0.18)<br>No mediation	0.03 (-0.35, 0.42)<br>No mediation	-0.05 (-0.33, 0.22)<br>No mediation	-0.04 (-0.33, 0.23)<br>No mediation	-0.02 (-0.09, 0.05)<br>No mediation	0.03 (-0.04, 0.10)<br>No mediation
Sugar (g/day)	**-0.08 (-0.15, -0.01)**<br>No mediation	-0.004 (-0.08, 0.07)<br>No mediation	-0.25 (-0.68, 0.19)<br>No mediation	0.09 (-0.29, 0.47)<br>No mediation	0.01 (-0.27, 0.29)<br>No mediation	0.11 (-0.16, 0.39)<br>No mediation	**-0.08 (-0.15, -0.004)**<br>No mediation	-0.03 (-0.10, 0.04)<br>No mediation

[a] Association between exposure and outcome: main effect presented when age is not a significant moderator. [b] Association between exposure and outcome not explained by mediators. [c] Association between exposure and outcome through mediator. [d] The proportion of the total effect mediated by mediator(s). All models were adjusted for age and individual-level SES and accounted for area-level clustering

Table 1B. Results of mediation analysis for the sub-sample, women (n=2607) and men (n=2264): total, direct, and indirect effects of area-level socio-economic status on mental health (outcomes). The Tromsø Study 2015-2016.

**Effects**	**Anxiety symptoms**		**Depression symptoms**		**Insomnia symptoms**	
	**Women**	**Men**	**Women**	**Men**	**Women**	**Men**
	B (95% CI)	B (95% CI)	B (95% CI)	B (95% CI)	B (95% CI)	B (95% CI)
Total effect[a]	-0.05 (-0.21, 0.11)<br>No significant interaction with age	0.04 (-0.10, 0.21)<br>No significant interaction with age	**-0.24 (-0.39, -0.09)**<br>No significant interaction with age	**-0.20 (-0.36, -0.04)**<br>No significant interaction with age	**-1.02 (-1.58, -0.46)**<br>No significant interaction with age	**-0.20 (-0.36, -0.04)**<br>No significant interaction with age
Direct effect[b]	-0.03 (-0.20, 0.11)<br>No significant interaction with age	0.07 (-0.08, 0.23)<br>No significant interaction with age	**-0.16 (-0.32, -0.02)**<br>No significant interaction with age	-0.13 (-0.29, 0.03)<br>No significant interaction with age	**-0.89 (-1.57, -0.40)**<br>No significant interaction with age	-0.13 (-0.29, 0.03)<br>No significant interaction with age
Indirect effect, combined[c]<br>PME[d]	-0.02 (-0.06, 0.04)<br>0.48 (-4.09, 4.49)	-0.03 (-0.07, 0.02)<br>-0.71 (-5.90, 5.63)	**-0.08 (-0.12, -0.02)**<br>0.33 (0.11, 0.86)	**-0.06 (-0.12, -0.02)**<br>0.33 (0.05, 1.37)	-0.12 (-0.22, 0.15)<br>0.12 (-0.07, 0.41)	**-0.06 (-0.12, -0.02)**<br>0.33 (0.05, 1.37)
Exposure-adjusted effects of mediators:						
Fruits and vegetables (g/day)	0.10 (-0.02, 0.21)<br>No mediation	0.009 (-0.09, 0.11)<br>No mediation	-0.09 (-0.19, 0.01)<br>No mediation	**-0.11 (-0.22, -0.006)**<br>Mediation	-0.04 (-0.42, 0.34)<br>No mediation	**-0.11 (-0.22, -0.01)**<br>Mediation
Saturated fat (g/day)	**0.33 (0.21, 0.44)**<br>No mediation	**0.23 (0.13, 0.33)**<br>No mediation	**0.20 (0.10, 0.30)**<br>No mediation	**0.16 (0.05, 0.26)**<br>No mediation	0.14 (-0.24, 0.53)<br>No mediation	**0.16 (0.05, 0.26)**<br>No mediation
Sugar (g/day)	**0.19 (0.08, 0.31)**<br>No mediation	**0.11 (0.01, 0.22)**<br>No mediation	**0.16 (0.05, 0.26)**<br>No mediation	0.07 (-0.04, 0.18)<br>No mediation	-0.07 (-0.46, 0.31)<br>No mediation	0.07 (-0.04, 0.18)<br>No mediation

[a] Association between exposure and outcome: main effect presented when age is not a significant moderator. [b] Association between exposure and outcome not explained by mediators. [c] Association between exposure and outcome through mediators. [d] The proportion of the total effect mediated by mediator(s). All models were adjusted for age and individual-level SES and accounted for area-level clustering

Tiwari S, *et al. J Epidemiol Community Health* 2024; 78:88–97. doi: 10.1136/jech-2023-220928

References

1. Hopstock LA, Løvsletten O, Johansen H, Tiwari S, Njølstad I, Løchen ML. Folkehelserapport. Den sjuende Tromsøundersøkelsen 2015-16. "Public health Report. The seventh Tromsø Study". Tromsø: UiT Norges Arktiske Universitet; 2019.
2. Tromsø kommune. Levekår i Tromsø: Geografisk fordeling. “living condition report”. Tromsø Municipality; 2019.
3. Sari E, Moilanen M, Bambra C, Grimsgaard S, Njølstad I. Association between neighborhood health behaviors and body mass index in Northern Norway: evidence from the Tromsø Study. Scandinavian Journal of Public Health. 2021:14034948211059972.
4. Tiwari S, Cerin E, Wilsgaard T, Løvsletten O, Njølstad I, Grimsgaard S, et al. Lifestyle factors as mediators of area-level socio-economic differentials in cardiovascular disease risk factors. The Tromsø Study. SSM - Population Health. 2022;19:101241.
5. Pallesen S, Bjorvatn B, Nordhus IH, Sivertsen B, Hjørnevik M, Morin CM. A new scale for measuring insomnia: the Bergen Insomnia Scale. Percept Mot Skills. 2008;107(3):691-706.
6. Snaith RP. The Hospital Anxiety And Depression Scale. Health Qual Life Outcomes. 2003;1:29.
7. Arntzen KA, Schirmer H, Wilsgaard T, Mathiesen EB. Impact of cardiovascular risk factors on cognitive function: the Tromsø study. Eur J Neurol. 2011;18(5):737-43.
8. Muller D, Judd CM, Yzerbyt VY. When moderation is mediated and mediation is moderated. J Pers Soc Psychol. 2005;89(6):852-63.
9. Cerin E, Conway TL, Adams MA, Barnett A, Cain KL, Owen N, et al. Objectively-assessed neighbourhood destination accessibility and physical activity in adults from 10 countries: An analysis of moderators and perceptions as mediators. Soc Sci Med. 2018;211:282-93.
10. Steen J, Loeys T, Moerkerke B, Vansteelandt S. Flexible Mediation Analysis With Multiple Mediators. Am J Epidemiol. 2017;186(2):184-93.

Tiwari S, *et al. J Epidemiol Community Health* 2024; 78:88–97. doi: 10.1136/jech-2023-220928
